# Buxton: Its Baths and Climate

**Published:** 1891-09

**Authors:** Alfred J. H. Crespi


### BUXTON: ITS BATHS AND CLIMATE.

BY

ALFRED J. H. CRESPI, M.R.C.S. Eng., M.R.C.P.I.

THE recent publication of an excellent book with this title, from the pen of Dr. Samuel Hyde, of Buxton, has directed much attention to a neighbourhood that in some respects has no rival in the United Kingdom. Buxton is in the very centre of the country, and stands higher above the sea-level than any other British town of moderate importance: on an elevated platform, thirty miles by fifteen, with a dry subsoil and comparatively rarefied air, it is the nearest approach we have to a mountain town, and is, in a far-away fashion, the English counterpart of Davos-Platz. True, it has a keen winter, much frost and snow, and a rather heavy rainfall—51 inches;—but its climate undoubtedly possesses features totally unlike those of any other familiar health-resort.

In one very important particular we Englishmen can challenge the world—we never know what is meant by a real drought. Sometimes we have what we are pleased to call long spells of dry weather, and the trees begin to look brown and faded, while the grass turns yellow and the roads are deep in dust. But would an American, a Spaniard, an Australian dignify a few rainless weeks as a drought? Some parts of our larger island never suffer from the modified drought which is at long intervals experienced on the south coast; and among the districts where the grass is always green and the trees always springlike in their freshness, the dales of Derbyshire take a foremost rank. Five years last summer the weather had been sufficiently sultry and dry, and our Dorset roads were much in need of rain, while the well-supply was running short and some pumps had quite given out. Late that August I went to Matlock, and what a marvellous contrast met my eye! The day of my arrival in Derbyshire was wet and the sky overcast, and I must confess that wetter weather I have no wish to see; but compensations there were—the verdure, which had deserted some other parts of the land, had taken refuge there, and I found a second and even more beautiful Spring. But Derbyshire dales are famous for something besides their perennial freshness:—for timber of magnificent dimensions; shade so thick that not even the foliage near Plymouth surpasses it; hills commonly fertile and well cultivated to their summits; sparkling rivers flowing merrily on, making sweet music as they go; and valleys which, when the sun shines upon them, are a foretaste of Paradise. Eliza Cook, in a few graceful lines, did full justice to this loveliness:

> "I was bound like a child by some magical story,
> Forgetting the South and Ionian vales,
> And felt that dear England had temples of glory
> Where any might worship in Derbyshire dales."

Had we only, in dear old England, three months of warm, sunny weather every year, who would care to wander to other lands? And the dales of Derbyshire would, during the summer, be crowded with visitors. Fortunately for lovers of rural solitude, our gloomy and uncertain climate drives many of our more fortunate countrymen to regions where the sun has greater power and shines with a persistency we never know, and so our inland and northern watering-places are seldom over-crowded.

South of Derby—at any rate near Birmingham—the country is not remarkable, being rather flat, not heavily timbered, and, though splendidly cultivated, refreshing only to fugitives from the crowded haunts of great cities, to whom any green field, any retired lane, is grateful. At Derby matters mend; but in spite of some pretty peeps, there is also a great deal of smoke, and the town, though clean and prosperous enough, is in parts dingy. As soon as Derby is left behind, the country becomes undulating, trees increase in size and number, the railway-cuttings are deeper, and the character of the scenery improves; and when we reach Ambergate, the junction for Sheffield, the woods, hills, and dales around prepare us for still greater beauties. Let us stop for a few hours at Matlock, cross the Derwent, and wander along the principal street of that most charming of watering-places: to our left rise richly-wooded heights; to our right stretch the irregularly-built but picturesque houses of Derwent Parade—town and country in actual juxtaposition, in Swiss fashion. From my childhood I had associated Buxton and Matlock with the most beautiful natural scenery; but circumstances did not admit of my paying a visit to them until five years ago. The weather in the district in which I live had been, as I have said above, extremely dry: for weeks we had had dust that would not have disgraced California; hedges, trees, grass, and gardens were turning brown; when, on the 26th of August, I slept at Derby, and next morning, before breakfast, ran on to Matlock and Buxton. The former was just awaking, and I had it pretty much to myself; but rain was descending in torrents that soon wetted me through and through. Such was my first visit. The wonderful verdure fascinated me; and the impression made has never been obliterated, though I have seen Matlock six times since, and generally under more propitious skies.

Going through Matlock a mile or so to Cromford, the rocks soon become grandly precipitous, rising 200 and 300 feet in almost perpendicular elevation. Cromford itself is mean, with only about a couple of hundred rather shabby houses, and a small unpretending church exquisitely hidden in a setting of fairy-like beauty. It has only one object of great interest, Willersly Castle, the residence of the Arkwrights. Should the road through Cromford be followed to Wirksworth, the latter is reached in an hour; but it offers little to interest: the church, generally the one large and beautiful building in an old English market-town, deserves no comment; while the newer streets, though in places broad and in marked contrast to the narrowness of some of the older carriage-ways, are sufficiently commonplace. A friend of mine (now with the majority) was Head Master of the Grammar School, and I often used to hear of the glories of Wirksworth and its neighbourhood; but my first visit, after my friend's removal to Sheffield, hardly came up to my expectation. Near Cromford, however, there is a walk, the Via Gellia, which for beauty, picturesqueness, and bold effects, makes a deep impression,—the Via Gellia runs along a lovely and retired valley a mile or more in length, and winds along at the foot of two ranges of stupendous rocks. The road was cut at the expense of Mr. Philip Gell, of Hopton, and has a certain resemblance to the Cheddar cliffs. The latter are loftier and more stupendous at their commencement, but they lack the fertility and beauty of the Via Gellia and do not extend a quarter so far. From Matlock Bath station a pretty road leads to Matlock Bridge; or the visitor may continue his journey by train, skirting the river. All the way from Matlock to Buxton the scenery is exquisite, and in bright sunshine, the peeps along the valley surpass anything I have seen elsewhere; while every mile or two to Miller's Dale, the little junction for Buxton, one passes places of historic interest—Darley Dale, Bakewell, Rowsley, Chatsworth, and Haddon Hall: while among the most noteworthy ecclesiastical buildings of the district, Tideswell Church is a noble edifice, and is the glory of that country-side; it is sometimes, on account of its immense size and great beauty, by a strange confusion of language, called the "Cathedral of the Peak."

From Miller's Dale to Buxton is about five miles. The country continues to rise gradually, and the vegetation tells of thinner soil and greater exposure; but the loveliness continues, and at intervals one sees stupendous masses of precipitous rocks, that would make the fortune of any other part of the country in attracting visitors; but in Peakland, where all is so beautiful, and deep valleys and lofty ridges meet the eye at every turn, no one thinks much of them.

On getting out at Buxton, the terminus of the branch, the visitor is struck by a departure from the usual order: towns, when natural shelter was important from scarcity of fuel and bad workmanship, used to be hidden away in deep, sheltered hollows; and it was not till the population increased, and the conveniences of life became commoner and greater, that buildings began to extend up the hills. But at Buxton there are two towns, which are rapidly being united: one—beautifully sheltered, richly wooded, and admirably built in a deep valley—is new; the other—of more antiquity and less pretension—is on higher ground and is freely exposed to the wind, and is not so heavily timbered; but residents maintain that, contrary to what one would expect, even Old Buxton is not much exposed, being effectually protected by the lofty hills surrounding it, which rise many hundreds of feet above every part of the town. New Buxton abounds in wealth and visitors. Its shops are handsome and filled with goods of excellent quality; while the streets, in favourable summer weather, are crowded with well-dressed and prosperous people, and it is not easy to imagine how so many thousands of persons can be stowed away.

Buxton is 33 miles north of Derby, and fully 1,100 feet above sea-level. The latter, in our latitude, is a great elevation, and more thoroughly changes the character of the vegetation than 4,000 feet in Southern Europe. Round the town the hills reach a height of 2,000 feet, and are said to be an impervious barrier to all but the strongest gales. The approach to the town is by a ravine, along which flows the Derbyshire Wye on its way to join the Derwent. Five miles to the east of the town is Chee Tor, an almost perpendicular limestone rock 400 feet above the Wye. The Romans discovered almost every natural spring which was likely to benefit invalids, and they did not overlook Buxton; they had baths there, and they seem to have frequented it for many generations: while as a modern watering-place, it has been a general favourite over three hundred years.

Among matters of historic interest, Mary Stewart stayed at Buxton for a time, when in the custody of Lord Shrewsbury; and all lovers of Scott will remember how closely *Peveril of the Peak*, his longest novel, is connected with this neighbourhood.

Thomas Hobbes, in 1678, when he was in his 91st year, published a Latin poem, *De Mirabilibus Pecci*, in which he described the wonders of the Peak in the quaint language of that age; and the healing properties of the waters were even then fully recognised:

> "Unto St. Anne the fountain sacred is;
> With waters hot and cold its sources rise,
> And in its sulphur-veins there medicine lies:
> This cures the palsied members of the old,
> And cherishes the nerves grown stiff and cold;
> *Crutches the lame unto its brink convey;*
> *Returning, the ungrates fling them away.*"

Some time earlier, Dr. John Jones wrote his well-known book, *The Bathes of Bathes Ayde*: it was published in 1572. This accomplished physician closely studied the composition and properties of the Buxton and the Bath waters, and seems to have divided his medical favours by practising sometimes in Somersetshire, sometimes in Nottinghamshire, and again in Derbyshire. In a book entitled *Buckstone's Bathes Benefyte*, published at the same time, Dr. Jones, speaking of the provision made for the accommodation of visitors to the Baths, anticipated the success which has attended the resort to those waters in modern times.

So that in the sixteenth century the reputation of the curative waters of Buxton was known far and wide, and many sufferers flocked to St. Anne's Well to be healed of divers painful maladies.

Poor Mary Queen of Scots paid repeated visits to Buxton to get such benefit as she could from its healing waters. Her first visit was in 1573, in the custody of the Earl of Shrewsbury.

Again, in 1576 and in 1580, we find her, still a prisoner in charge of the Earl of Shrewsbury, visiting Buxton. In a letter to Lord Burleigh, dated August 9th, 1580, the Earl writes:

"I cam heddar to Buxtons w$^{t}$ my charge, the 28 of July. She hadde a harde begynnenge of her jorney; for when she shuld have taken her horse, he started asyde, and therwith she fell and hurte her bake, w$^{ch}$ she still complaines off, notwithstanding she applyes the bathe ons or twyse a daye. I doo strictly obsarve hur Ma$^{ties}$ com'andment, wrytten to me by yo$^{r}$ L. in restreyninge all resorte to this place; nether dothe she see, nor is seene to any more than to hur owne pepell and such as I appoynt to atende: she hath nott come forthe of the house synce her cumynge, or shall nott before hur p'tynge."

The condition of poor Mary, sick in body and broken in spirit, was very sad. She found relief for her pent-up feelings on her last visit, in 1582, by writing on a pane of glass in her room the following Latin lines:

"*Buxtona, quæ celebrabere nomine lymphæ,*

*Forte mihi posthac non adeunda, vale!*"

which have been somewhat freely translated thus:

"Buxton, whose fame thy milk-warm waters tell,
Whom I, perhaps, no more shall see, farewell!"

It is pleasant to think that her sufferings were relieved, for she wrote that: "It is incredible how the water has relaxed the tension of the nerves, and relieved my body of the dropsical humours with which, in consequence of my debility, it has been charged."

The springs supplying the baths are calcareous and chalybeate. The former are mostly tepid, with a temperature of 82° F., but some are quite cold. During the season, which now only lasts from June to October, the place is visited by many thousands, from all parts of the kingdom (and Dr. Hyde says, *of the world*), who make it their residence for a time; while enormous crowds of day excursionists go over from Derby and Nottingham, and some from even more distant places, such as Birmingham, Wolverhampton, Liverpool, and Manchester. Buxton is a convenient starting-point for excursions, and of late years it has been made very easy of access from all parts of the country. The waters are being increasingly used in indigestion, gout, rheumatism, and skin affections. The accommodation for visitors is large, and 4,000 at a time can be comfortably lodged. Like Ilfracombe, Bournemouth, and Matlock, it is mainly a town for invalids, with every convenience and attraction for the sick. It is justly proud of the Devonshire Hospital, its most famous public institution, which contains a hundred beds and receives nearly a thousand patients yearly, who are boarded and treated gratuitously. The rise and magnificence of Buxton is greatly owing to the Dukes of Devonshire. One duke, in the last century, spent £120,000 erecting, close to the site of the railway terminus and between the towns, The Crescent, an immense three-storied range of buildings of gritstone. The main part of this is a vast curve, extending 200 feet, with wings 58 feet long. It contains two large hotels, a library, and some handsome shops.

Mr. Edward Bradbury, in a recently-published work called *Pictures of the Peak*, gives a charming description of Buxton in the last century. I take the following passages:

"Some places are analogous with people. They have their youth and manhood, their ripe old age and tottering decay. As with the individual, so with the town. It is a story of rise and progress, prosperity and opulence, decline and fall. Some towns reverse this rule. As they grow older they become younger, and advancing years only bring with them higher spirits, brighter looks, increased power, and more vivacious vitality. Buxton was a place of established reputation before the existence of some modern cities, and when great manufacturing centres, with their teeming, toiling populations, remained unreclaimed from moss and marsh and moor. Yet Buxton is younger to-day than it was when Mary Queen of Scots counted her rosary at the Old Hall, and those foppish courtiers, Leicester and Burleigh, in slashed and puffed doublets, velvet cloaks and jewelled belts, drank the waters and strutted with swaggering gait on the bowling-green. Two thousand years ago, the Spa of the Peak was in its teens. Unsightly tenements have been replaced by handsome buildings, and unsavoury alleys by spacious thoroughfares. Some picturesque old edifices have been demolished—quaint, half-timbered, latticed-windowed, high-gabled bits of architecture, grey with age, and wrinkled with russet reminiscences—that an artist would have spared. But comfortable cottages have succeeded cave-dwellings, and tumble-down houses have been improved off to make way for elegant villas and imposing terraces, great hotels and halls of health. An old, grubby limestone chrysalis has been metamorphosed into the brightest of architectural butterflies. The translation has been a protracted one, for the Peak district is famous for its 'fossils,' and the petrifying process sometimes congeals mind as well as matter. In the reign of Beau Nash, Bath was a more fashionable residential place than Buxton is in the ninth decade of the nineteenth century, and its buildings were quite as pretentious, thanks to the genius of Wood, an architect who made artistic use of natural advantages. In the time of the Tudors, however, Bath was more celebrated for its wool than its waters; while Buxton was the Mecca of pilgrims from all parts, who were attracted by the medical fame of St. Anne's Well, and who left their crutches at the shrine in evidence of the relief which they had found from their sufferings. But it is not much more than a hundred years since Buxton really emerged from an uncouth moorland village into an aristocratic watering-place.

"The Pump Room of a hundred years ago compares very favourably with the shabby apology for such an institution which exists to-day. The springs bubbled up in sparkling purity into glistening marble basins, and there was an absence of the vulgar public-house beer-handles that decorate the counter of the present meagre

14

apartment, which is deemed good enough to receive the visitor accustomed to the superb pump-rooms and Kursaals of the Continent. St. John's Church, with its *hôtel-de-ville*-like exterior and Turkish-bath-like ceiling, was not erected until 1812. A century ago the hotels competed with the tottering Chapel of St. Anne's in offering spiritual edification to Buxton visitors. A clergyman—the Rev. Mr. Thorpe—attended daily at the Old Hall, saying grace and reading prayers. He was well paid for his pious pains. 'As a reward for his trouble, when the cloth is removed, and he has left the room, the principal gentleman at the table goes round with the plate to the company, who give what they please; never less than a shilling, and a few more a *piece*, which amounted generally to four pounds.' Think of that, churchwardens of St. John's and St. James's, when all the preaching of the most powerful pulpiteers cannot extricate your funds from debt! But it was not all sermons and soda-water at Buxton a hundred years ago. The visitors gambled as much as they did at Bath during the Regency, and the preponderance of people who subscribed to the Card Room over those who supported the Church, gave rise to the satirical lines:

'The Church and Rooms the other day
Open'd their books for prayer and play:
The priest got *twelve*, Hoyle sixty-seven;
How great the odds for Hell 'gainst Heaven!'

"There were amusements in plenty in 1788. The Duke of Devonshire surpassed Mæcenas in the opulency of his patronage. He provided a private band at his personal cost. The races were permanently fixed for the Wednesday and Thursday following the first Sunday after Trinity. A subscription pack of harriers was kept in the neighbourhood. The best London talent appeared at the Theatre, for the metropolitan playhouses were closed during the Buxton season. The performance commenced at six o'clock, the latest hour for *table d'hôte* being four o'clock. The Assembly Room was the scene of aristocratic dances. The White Hart sent its respectful compliments to the Angel, and asked its company to its dance after supper. Other inns exchanged mutual courtesies of this description. There were no bath-chairs: it was the age of sedan-chairs. Oil lamps in the streets made the darkness visible. Linkmen with flaring torches were the escort of ball parties. The Assembly Room was a blaze of wax-candles in crystal chandeliers. Wandering about the Crescent in the moonlight, I peopled the eloquent place with the phantoms of the past; with crush of fashion and galaxy of beauty; with Youth and Hope and Dreams; with Avarice and Hate and Despair; with Love and Lust, Ambition and Failure; with Lady Teazle and Joseph Surface, Sir Benjamin Backbite and Lady Sneerwell, tender Maria and honest Rowley; with wigs and patches *à la Grecque*, hoops and red-heeled shoes, glistening sword-hilts and cocked-hats. The sedan-chairs set down radiant beauties with powdered hair and jewelled dresses, chaperones and dowagers, fine old 'bucks' and gallant beaux with marvellous wigs, coats of Tyrian bloom, bravely embroidered waistcoats, and garter-blue-silk breeches and dangling swords. The windows send yellow shafts of light across the roadway. Music floats in the night air. It is a stately minuet. There is a ripple of laughter in the intervals, and everybody seems to be talking to everybody else. The music starts again, and there is the *frou-frou* of silk and brocade on the ball-room floor. I should like to join the dancers; but I must first go home and array myself in a full-skirted murrey-coloured coat, don a flowered waistcoat, encase my legs in breeches of sarsenet, put on silk stockings, comb my bag-wig, and further adorn my person with fine lace ruffles and a jabot. But what is the use of these vanities? The gay company upstairs have long gone home. It is only the wandering shadows of a hundred years ago that haunt the Crescent to-night."

A radical change has come over English society: the aristocratic and opulent no longer desert London *en masse* for any provincial town and make it their residence for many months. With tenfold more travelling and twenty times more money than a century and a half ago, people of quality run to any place that takes their fancy at almost any season—it may be this year going to Scotland, next to Italy, the year after to some unheard-of hamlet on the Cornish or the Norfolk coast, and the longer journeys are filled in with nearly twice as many short ones. Watering-places, though more prosperous than ever, are no longer what they once were: society no longer migrates to Tunbridge Wells or Bath, Cheltenham or Buxton; and if perchance a few peers and their families stay half a dozen weeks in one of these places, there is nothing in their habits, appearance, or dress to distinguish them from other educated, affluent people. But though the people have altered, the natural features remain unchanged, and Buxton and Matlock grow on one as do few other places.

Near Buxton is Diamond Hill, remarkable for its crystals; and Poole's Hole, a stalactite cavern a quarter of a mile long. The Baths, too, which were rebuilt some years ago, are among the finest in Europe. With all these natural and artificial attractions, it is not surprising that the population of the town and its suburbs is growing fast, and increased from 3,717 in 1871 to nearly 7,000 in 1881, and must now considerably exceed 10,000; while building continues in all directions.

During my last visit, unfortunately far too brief and hurried, I was the guest of my friend Dr. Hyde, who, besides the more pretentious work which gives its name to this article, is the author of a smaller but very useful book called *Peakland*. Dr. Hyde is the resident physician of the Peak Hydropathic Establishment, which has lately been greatly enlarged, and is now fitted up with the most perfect modern contrivances. In proof of what Buxton can offer in the way of comforts and advantages, and as showing the progress which modern establishments for the relief of invalids are making, I cannot do better than describe the arrangements. The house is lighted throughout by the incandescent electric light; it has a hydraulic passenger-lift, and it is warmed in every part with hot water. It is boldly situated on rising ground, only five minutes from the station; and it is also close to the public gardens, the shops, and the baths. It has a magnificent drawing- and music-room, one hundred feet long; the table is well supplied, and the six o'clock dinner is a model of what a public *table d'hôte* should be, while the charges are moderate when allowance is made for the sumptuousness of the entertainment. Inmates in Water-Cure establishments are no longer subjected to the very severe regimen and harsh treatment of half a century ago. Many of the visitors, indeed, are in perfect health, and accompany friends; others prefer the lively society and good sanitary arrangements of such a home, to private lodgings: so that there is nothing depressing in a residence in them.

Health-resorts depend for their prosperity absolutely on the perfection and cheapness of their boarding-houses, hotels, and hydropathic establishments, and in few places is there less room for improvement than at Buxton; indeed, it seems impossible to make important changes, for everything is as good as can be—the *ne plus ultra* of perfection is almost reached.

The air of Buxton is sharp and often cold—that follows from the inland situation of the town and its great elevation—and comparatively few people would choose it as a winter residence. Dr. Hyde is nevertheless making strenuous efforts to give it a winter as well as a summer season, and is urging the residents to second him in his undertaking; and he begs them not to talk of "the season," but of the summer and of the winter seasons. At first sight, it hardly looks as though he could succeed. If there is one thing that invalids dread, it is cold and gloom, and it would greatly surprise me if they deserted Ilfracombe, Falmouth, Jersey, and Bournemouth for a town six to eight degrees colder in mid-winter, and with much less sunlight at all seasons. Nevertheless, Dr. Hyde, whose ability and experience are beyond dispute, stoutly maintains that he is right, and that Buxton ought to have a prosperous winter season; for the cold is quite bearable and the air fresh and invigorating in ordinary winters. Indeed, he argues that it is an excellent winter station in early consumption. Some cases may be benefited by the cold upland air, and experience will show which they are. Certainly invalids from warmer and more sheltered regions sometimes speak gratefully in its praise. A gentleman from Wimborne, who was in the neighbourhood of Buxton in the late autumn, a few years ago, tells me that he enjoyed his ante-breakfast walk wonderfully, though there was no dearth of snow and frost.

It should not be overlooked that great part of the benefit of a residence in such a town is due to the complete change and rest; so that, in spite of a less genial winter than that of the south and west coasts, a few weeks in a well-found hydropathic establishment of the first order, or in some nicely-managed boarding-house, may be a real treat: and it is something to be surrounded by fresh faces, and to hear unaccustomed voices.

The mean temperature of the year is 45.2°; while that of Greenwich is 49.12°, and that of Barnstaple, Falmouth, and Helston, 51.5°. Buxton is, therefore, over 6° colder, while in the matter of sunlight it has barely half the number of hours to be counted upon at Wimborne, Romsey, and Hastings. Dr. Hyde, however, says that the colder the season the greater the proportion of recoveries, and the more decided the improvement in the condition of visitors. This, he adds, was especially seen in the summer of 1888, when patients did much better than in 1887. Not being an invalid, I can hardly speak with authority; though I am ready to confess that had I leisure and means, there are few places which I should choose with greater delight than Buxton and Matlock for a brief holiday. Never, however, being fortunate enough to have any holidays, I can only speak of Buxton as I have found it when I have been called there on professional engagements; and as a place attractive to a degree, with an indescribably beautiful neighbourhood, it seems to me to deserve far more attention than it has yet received.

